# Lipocalin 2 prevents intestinal inflammation by enhancing phagocytic bacterial clearance in macrophages

**DOI:** 10.1038/srep35014

**Published:** 2016-10-13

**Authors:** Takahiko Toyonaga, Minoru Matsuura, Kiyoshi Mori, Yusuke Honzawa, Naoki Minami, Satoshi Yamada, Taku Kobayashi, Toshifumi Hibi, Hiroshi Nakase

**Affiliations:** 1 Center for Advanced IBD Research and Treatment, Kitasato University Kitasato Institute Hospital, 5-9-1, Shirokane, Minato-ku, Tokyo, 108-8642, Japan; 2 Department of Gastroenterology & Hepatology, Graduate School of Medicine, Kyoto University, 54 shogoin, Kawahara-cho, Sakyo-ku, Kyoto, 606-8397, Japan; 3 School of Pharmaceutical Sciences, University of Shizuoka, 52-1, Yada, Suruga-ku, Shizuoka, 422-8526, Japan; 4 Department of Gastroenterology and Hepatology, Sapporo Medical University School of Medicine, S-1, W-16, Chuo-ku, Sapporo, 060-8543, Japan.

## Abstract

Lipocalin 2 (Lcn2), also called neutrophil gelatinase B-associated lipocalin (NGAL), is an anti-microbial peptide originally identified in neutrophil granules. Although Lcn2/NGAL expression is increased in the inflamed intestinal tissues of patients with inflammatory bowel disease, the role of Lcn2/NGAL in the development of intestinal inflammation remains unclear. Here we investigated the role of Lcn2/NGAL in intestinal inflammation using a spontaneous mouse colitis model, interleukin-10 knock out (IL-10 KO) mice. Lcn2 expression in the colonic tissues of IL-10 KO mice increased with the development of colitis. Lcn2/IL-10 double-KO mice showed a more rapid onset and development of colitis compared to IL-10 KO mice. Lcn2 enhanced phagocytic bacterial clearance in macrophages *in vitro* after infection with *Escherichia coli*. Transfer of Lcn2-repleted macrophages prevented the development of colitis in Lcn2/IL-10 double-KO mice *in vivo*. Our findings revealed that Lcn2 prevents the development of intestinal inflammation. One crucial factor seems to be the enhancement of phagocytic bacterial clearance in macrophages by Lcn2.

Inflammatory bowel disease (IBD) is a chronic relapsing-remitting disorder characterized by recurrent intestinal inflammation. Although the pathophysiology of IBD remains unclear, intestinal inflammation appears to result from a dysregulated mucosal immune response toward enteric commensal bacteria and their interactions with environmental triggers, including dietary elements^1^. The role of specific food components or nutrients in the pathophysiology of IBD is uncertain, but findings from recent clinical and experimental studies suggest that dietary iron contributes to the development of IBD by its effects on enteric bacterial flora^2^,^3^,^4^.

Lipocalin 2 (Lcn2), also called neutrophil gelatinase B-associated lipocalin (NGAL), is a member of the lipocalin superfamily of extracellular transport proteins^5^. In addition to the function of lipocalins as transporters of some lipophilic molecules, such as retinoids, fatty acids, and cholesterol, Lcn2/NGAL is involved in iron delivery, cell migration, apoptosis, and cell differentiation^6^,^7^,^8^,^9^. One of the best-characterised functions of Lcn2/NGAL is to deprive bacteria of iron essential to their growth by sequestering iron-laden siderophores, a diverse group of small non-peptide iron-binding chemicals produced by bacteria^6^,^10^. Indeed, Lcn2-deficient mice are more sensitive to bacterial infection than wild-type mice, and exhibit higher mortality rates after intraperitoneal challenge with *Escherichia coli*^11^. In this regard, Lcn2/NGAL plays an essential role in the innate immune response against bacterial infection.

While Lcn2/NGAL was originally identified as a component of neutrophil granules, it is also expressed in macrophages and epithelial cells of the respiratory and gastrointestinal tracts in response to inflammatory signals^12^,^13^,^14^,^15^. Lcn2/NGAL expression is increased in the colonic tissues of patients with active IBD^16^,^17^. Furthermore, Lcn2/NGAL levels are increased in the serum, urine, and feces of patients with active IBD^18^,^19^,^20^. The precise role of Lcn2/NGAL in IBD pathophysiology, however, remains to be elucidated. Therefore, in the present study, we investigated the role of Lcn2/NGAL in intestinal inflammation using a spontaneous mouse colitis model, interleukin-10 knock out (IL-10 KO) mice.

## Results

### Lcn2 expression in the colonic tissues of IL-10 KO mice increased with the development of colitis

**Figure 1:**
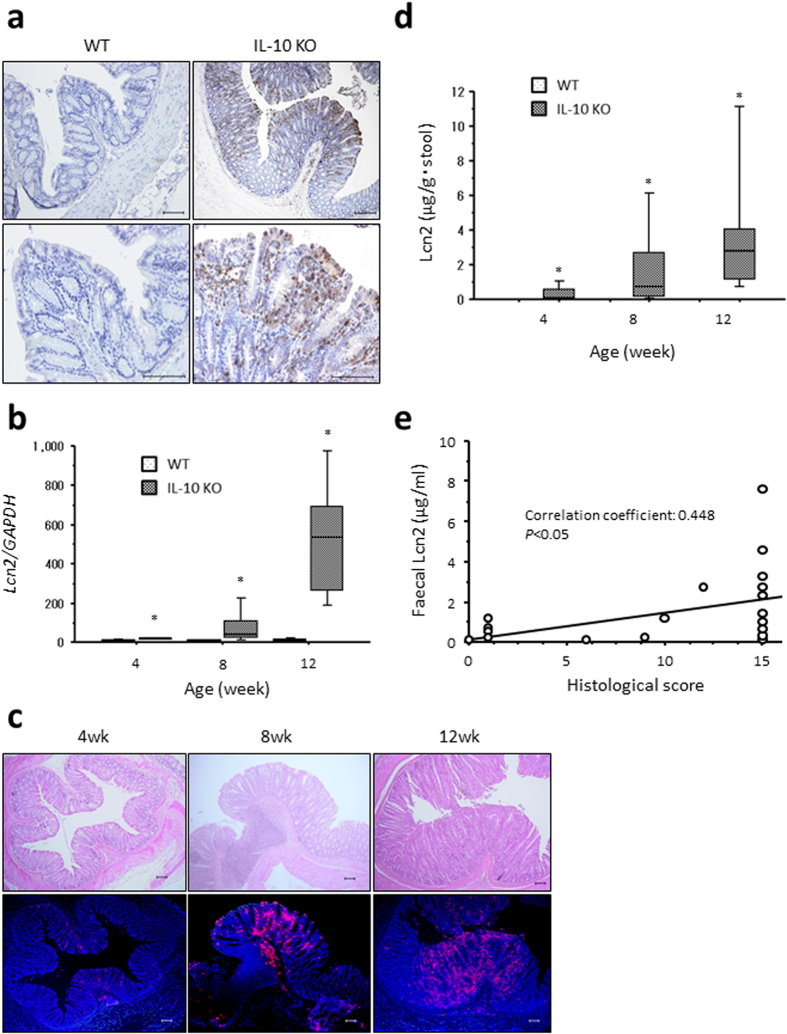
Increased Lcn2 expression in the colon of IL-10 KO mice. (**a**) Representative images of Lcn2 expression in the colonic tissues of WT and IL-10 KO mice at 12 weeks of age. Scale bars, 100 μm. (**b**) Gene expression of *Lcn2* in the colonic tissues of WT and Lcn2 KO mice. N = 8 for each group at the indicated age (in weeks). The box refers to the interquartile range and the bar inside represents the median. *P < 0.05 compared with WT mice by non-parametric Mann-Whitney U test. (**c**) Representative images showing Lcn2 expression in the colonic tissues of WT and Lcn2 KO mice at the indicated age (in weeks). Upper panels, HE staining. Lower panels, immunofluorescent staining of Lcn2 (red) counterstained with 4′,6-diamidino-2-phenylindole (blue). Scale bars, 100 μm. wk, week of age. (**d**) Faecal Lcn2 concentrations in WT and IL-10 KO mice at the indicated ages (in weeks). N = 8 for each group. The box refers to the interquartile range and the bar inside represents the median. *P < 0.05 compared with WT mice by non-parametric Mann-Whitney U test. (**e**) Correlation between fecal Lcn2 and histological score in IL-10 KO mice assessed by the Spearman correlation coefficient test.

Lcn2 expression in the colonic tissues of 12-week-old C57BL/6 wild-type (WT) and IL-10 KO mice was evaluated by immunohistochemical analysis. Lcn2 expression was observed mainly in the epithelial cells and partly in infiltrating immune-cells in the colonic tissues of IL-10 KO mice, whereas little Lcn expression was detected in WT mice (Fig. 1a). Gene expression of *Lcn2* in the colonic tissues of IL-10 KO mice increased in a time-dependent manner (Fig. 1b). Similarly, immunohistochemical analysis revealed that Lcn2 expression increased in the colonic tissues of IL-10 KO mice over time (Fig. 1c). Furthermore, the fecal concentration of Lcn2 in IL-10 KO mice remarkably increased within a time interval of 12 weeks of colitis induction in the IL-10 KO mice (Fig. 1d) and was significantly positively correlated with the degree of histological inflammation (Fig. 1e).

### Both IL-1β and Toll-like receptor 4 signaling pathways play a crucial role in enhancing Lcn2 expression in the inflamed colonic tissues of IL-10 KO mice

**Figure 2:**
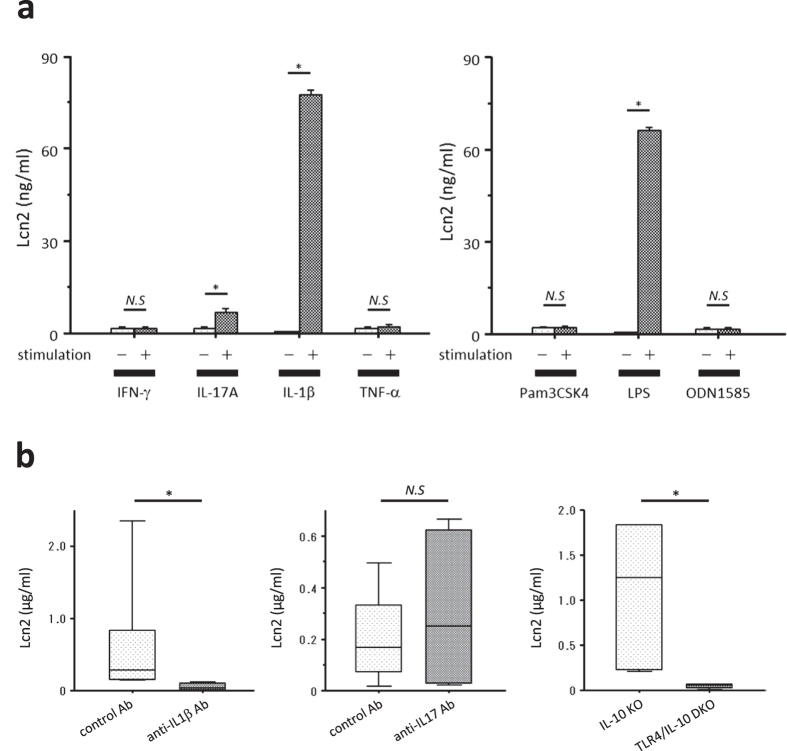
Involvement of IL-1β and TLR4-signaling pathways in enhanced Lcn2 expression *in vitro* and *in vivo*. (**a**) Lcn2 secretion from CT26 cells after stimulation with or without IFN-γ (10 ng/ml), IL-17A (200 ng/ml), IL-1β (10 ng/ml), TNF-α (100 ng/ml), Pam3CSK4 (1 μg/ml), LPS (100 ng/ml), and ODN1585 (100 nM). N = 6 for each group. Error bars represent SEM. (**b**) Effect of neutralizing antibodies against IL-1β and IL-17, and TLR4 deficiency on fecal Lcn2 concentrations in IL-10 KO mice at 4 weeks of age. N = 5 per group. The box refers to the interquartile range and the bar inside represents the median. *P < 0.05 compared with non-stimulated group (**a**) or control antibody group (**b**) by non-parametric Mann-Whitney U test. Ab, antibody. *N.S.*, not significant.

To reveal how Lcn2 expression is regulated in the colonic epithelia, we performed an *in vitro* experiment using a murine colonic epithelial cell-line, CT26. Stimulation with IL-17A and IL-1β significantly increased Lcn2 secretion from CT26 cells, whereas stimulation with interferon-gamma (IFN-γ) and tumour necrosis factor-alpha (TNF-α) did not (Fig. 2a). In addition, lipopolysaccharides (LPS), a ligand of Toll-like receptor (TLR) 4, significantly increased Lcn2 secretion from CT26 cells, whereas ligands for TLR2 and TLR9 did not (Fig. 2a).

To confirm the influence of these stimulants *in vivo*, we examined the effect of neutralizing antibodies against IL-17 and IL-1β on the fecal concentration of Lcn2 in IL-10 KO mice. Administration of anti-IL-1β antibody, but not anti-IL-17 antibody, significantly decreased the fecal Lcn2 concentration in IL-10 KO mice at 4 weeks of age (Fig. 2b). We also examined the effect of TLR4 deficiency on the fecal concentration of Lcn2 in IL-10 KO mice using TLR4/IL-10 DKO mice. Fecal Lcn2 concentrations were significantly lower in TLR4/IL-10 DKO mice than in IL-10 KO mice at 4 weeks of age (Fig. 2b).

### Lcn2 deletion elicits spontaneous colitis in IL-10 KO mice at an early age

**Figure 3:**
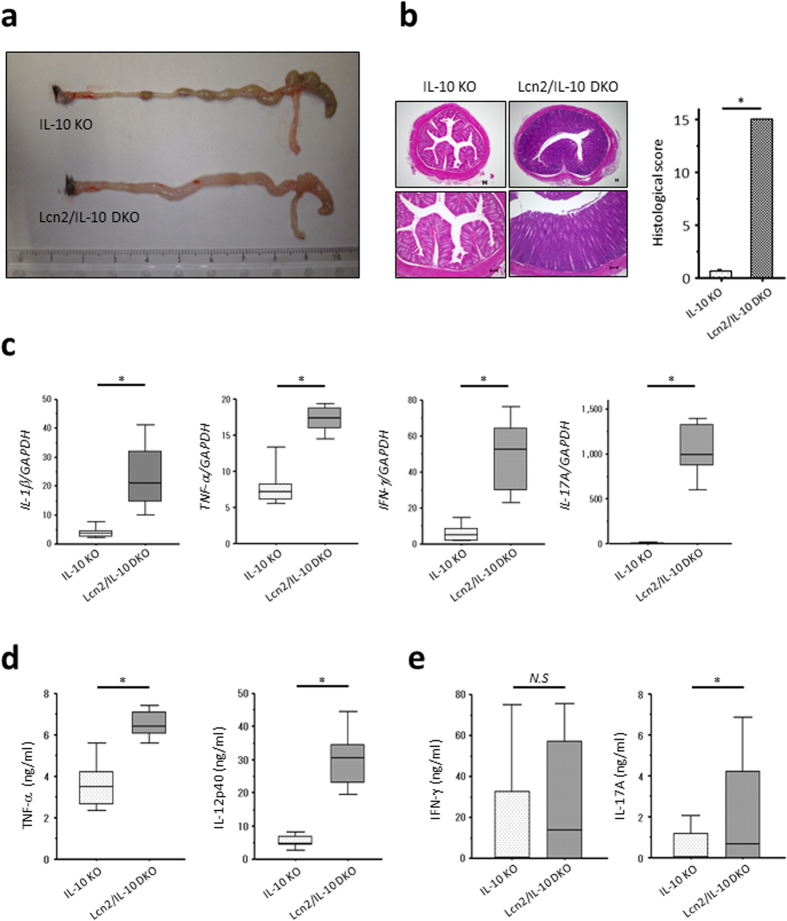
Accelerated onset of colitis in Lcn2/IL-10 DKO mice compared to IL-10 KO mice. (**a**) Representative macroscopic findings of the colons of IL-10 KO and Lcn2/IL-10 DKO mice at 4 weeks of age. (**b**) Representative HE-staining images (left) and histological scores (right) of the colonic tissues of IL-10 KO and Lcn2/IL-10 DKO mice at 4 weeks of age. Scale bars, 100 μm. N = 9 for each group. Error bars represent SD. *P < 0.05 compared with IL-10 KO mice by non-parametric Mann-Whitney U test. (**c–e**) Gene expression of *IL-1*β, *TNF-*α, *IFN-*γ, and *IL-17A* in the colonic tissues (**c**), TNF-α and IL-12p40 secretion from colonic explants (**d**), and IFN-γ and IL-17A secretion from unseparated MLN cells stimulated with CBLs (**e**), of IL-10 KO and Lcn2/IL-10 DKO mice at 4 weeks of age. Gene expression of each target molecule was normalized to *GAPDH*. N = 7–8 per group. The box refers to the interquartile range and the bar inside represents the median. *P < 0.05 compared with IL-10 KO mice by non-parametric Mann-Whitney U test. MLN, mesenteric lymph-node. CBLs, cecal bacterial lysates. *N.S.*, not significant.

To assess the role of Lcn2 in intestinal inflammation, we compared intestinal inflammation between IL-10 KO and Lcn2/IL-10 DKO mice, because Lcn2 KO mice did not develop spontaneous colitis (Supplementary Fig. 1). Macroscopically, remarkable shrinkage of the cecum and stiffening of the colonic wall were observed in Lcn2/IL-10 DKO mice as early as 4 weeks of age (Fig. 3a). Similarly, histological scores were significantly higher in Lcn2/IL-10 DKO mice than in IL-10 KO mice, even at 4 weeks of age (Fig. 3b). Gene expression of pro-inflammatory cytokines, such as *IFN-*γ, *IL-17A*, *IL-1*β, and *TNF-*α, was significantly upregulated in the colonic tissues of Lcn2/IL-10 DKO mice compared with IL-10 KO mice (Fig. 3c). Also, colonic explants from Lcn2/IL-10 DKO mice secreted significantly higher levels of TNF-α and IL-12p40 than those from IL-10 KO mice (Fig. 3d). Mesenteric lymph node (MLN) cells from Lcn2/IL-10 DKO mice produced greater amounts of IL-17A than MLN cells from IL-10 KO mice after stimulation with cecal bacterial lysates (CBLs; Fig. 3e).

### Lcn2 deficiency does not affect mucosal barrier function

The enhanced Lcn2 production in the colonic epithelia of IL-10 KO mice implied the involvement of Lcn2 in mucosal barrier function. Thus, we examined the effect of Lcn2 deficiency on enteric bacteria by comparing enteric bacterial flora between WT and Lcn2 KO mice. Terminal restriction fragment length polymorphism (T-RFLP) analysis, however, revealed no significant difference in the luminal bacterial composition between WT and Lcn2 KO mice at 4 weeks of age (Supplementary Fig. 2a, Supplementary Table 1). We also examined the expression of intestinal barrier-related molecules in the colonic tissues of WT and Lcn2 KO mice. The number of Ki-67 positive colonic epithelial cells (Supplementary Fig. 2b) and *HES-1* gene expression in the colonic tissues (Supplementary Fig. 2c) did not differ significantly between WT and Lcn2 KO mice. Moreover, expression of TLR genes (*TLR2*, *TLR4*, and *TLR9*), junctional molecules (*ZO-1* and *Claudin-1*), mucin (*MUC2*), trefoil factor (*TFF3*), and anti-microbial peptides (*mBD3*, *CRAMP*, and *REG3*γ) in the colonic tissues was not significantly different between WT and Lcn2 KO mice (Supplementary Fig. 2d).

### Lcn2 affected phagocytosis and intracellular bacterial clearance in macrophages

**Figure 4:**
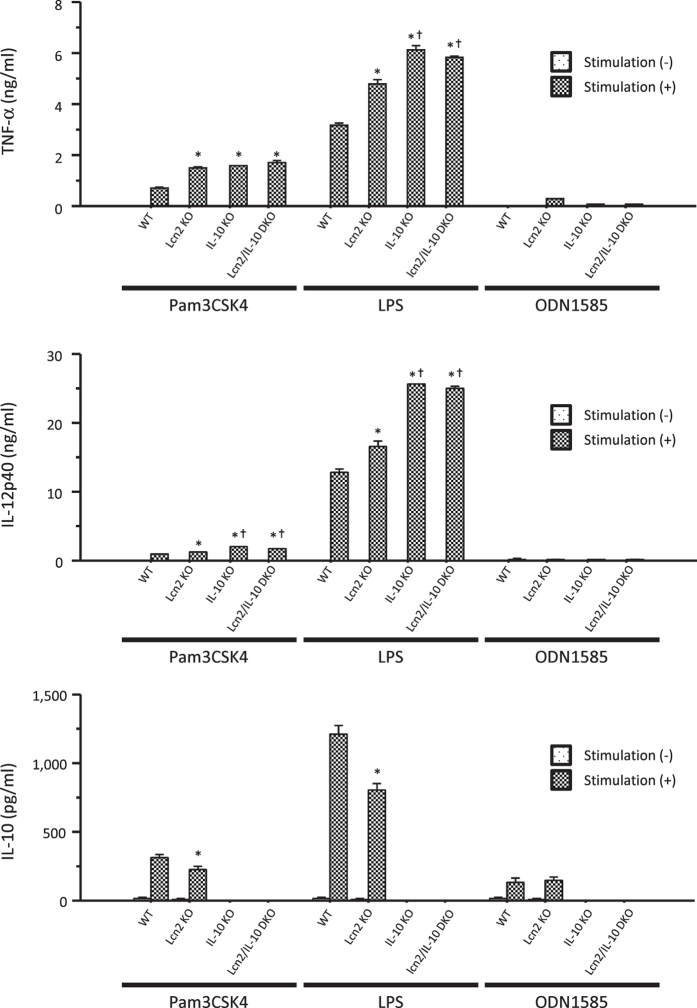
Effect of Lcn2 deletion on cytokine production from macrophages. Cytokine production from thioglycollate-elicited peritoneal macrophages (TEPMs) stimulated with or without Pam3CSK4 (500 ng/ml), LPS (100 ng/ml), and ODN1585 (500 nM) for 24 hours. N = 6 per group. Error bars represent SEM. *P < 0.05 compared with WT mice; ^†^*P* < 0.05 compared with Lcn2 KO mice by one-way ANOVA with Bonferroni’s correction.

To investigate the role of Lcn2 in mucosal innate immunity, we next examined the effect of Lcn2 deficiency on macrophage functions using thioglycollate-elicited peritoneal macrophages (TEPMs) obtained from WT (WT-derived TEPMs), IL-10 KO (IL-10KO-derived TEPMs), Lcn2 KO (Lcn2KO-derived TEPMs), and Lcn2/IL-10 DKO mice (DKO-derived TEPMs). Lcn2KO-derived TEPMs secreted significantly higher amounts of TNF-α and IL-12p40 than WT-derived TEPMs after stimulation with Pam3CSK4 and LPS, while they secreted significantly lower amounts of IL-10 than WT-derived TEPMs. The levels of these pro-inflammatory cytokines did not differ significantly between IL-10KO and DKO-derived TEPMs (Fig. 4).

**Figure 5:**
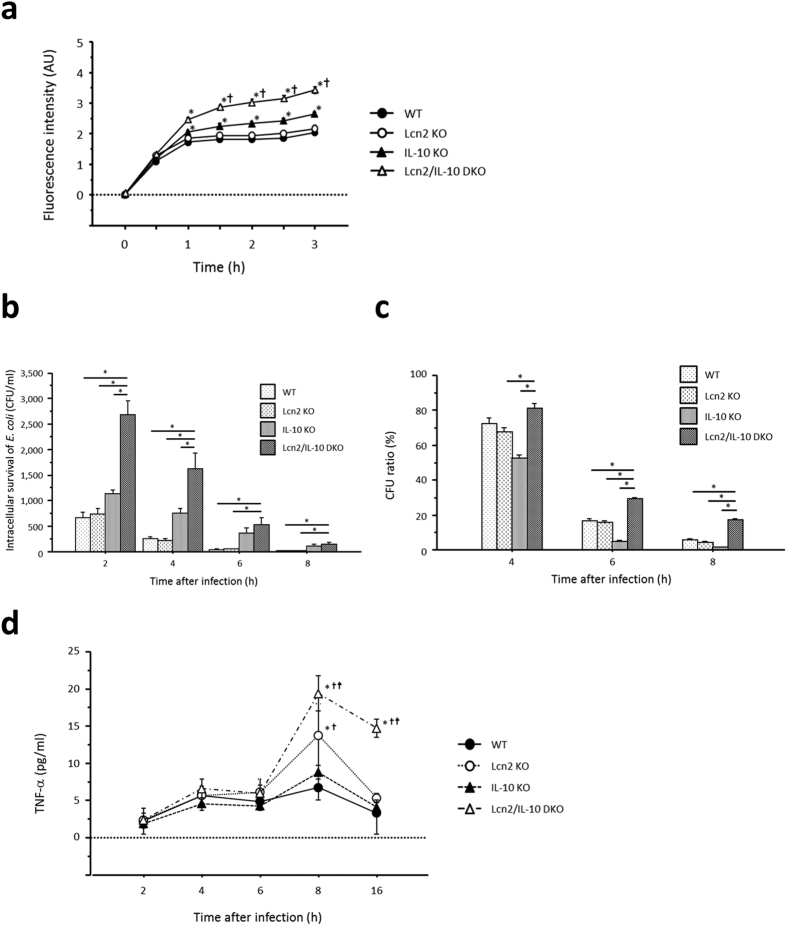
Involvement of Lcn2 in phagocytosis and intracellular bacterial clearance in IL-10 KO mouse-derived macrophages. (**a**) Time-dependent changes in fluorescence intensity in thioglycollate-elicited peritoneal macrophages challenged with fluorescent particle conjugated-*E. coli*. Fluorescence intensity was measured at the indicated times and is shown in arbitrary units (AU). N = 9 per group. Error bars represent SEM. *P < 0.05 compared with WT mice; ^†^*P* < 0.05 compared with IL-10 KO mice by one-way ANOVA with Bonferroni’s correction. (**b**) Intracellular survival of *E. coli* in macrophages at the indicated times after infection. N = 6 per group. Error bars represent SEM. *P < 0.05, one-way ANOVA with Bonferroni’s correction. (**c**) Ratio of colony forming units (CFU) at the indicated times to CFU at 2 hours after *E. coli* infection. N = 6 per group. Error bars represent SEM. *P < 0.05, one-way ANOVA with Bonferroni’s correction. (**d**) TNF-α secretion from macrophages after infection with *E. coli*. N = 6 per group. Error bars represent SEM. *P < 0.05 compared with WT mice; ^†^*P* < 0.05 compared with IL-10 KO mice; ^‡^*P* < 0.05 compared with Lcn2 KO mice by one-way ANOVA with Bonferroni’s correction.

The phagocytosis assay revealed enhanced phagocytosis of *E. coli* in DKO-derived TEPMs beginning 1 hour after challenge with *E. coli* compared to IL-10KO-derived TEPMs (Fig. 5a). Furthermore, intracellular survival of phagocytosed *E. coli* in DKO-derived TEPMs persisted compared to that in IL-10KO-derived TEPMs (Fig. 5b,c). DKO-derived TEPMs produced significantly higher amounts of TNF-α than other TEPMs after infection with *E. coli* (Fig. 5d). In contrast to IL-10KO-derived TEPMs, Lcn2 deficiency had no effect on either phagocytic function or intracellular bacterial clearance of WT-derived TEPMs. Therefore, we compared Lcn2 expression between WT- and IL-10KO-derived TEPMs after infection with *E. coli*. IL-10KO-derived TEPMs, but not WT-derived TEPMs, expressed Lcn2 after 1-hour infection with *E. coli* (Supplementary Fig. 3).

### Lcn2 enhanced phagocytotic/autophagic clearance of *E. coli* in macrophages

**Figure 6:**
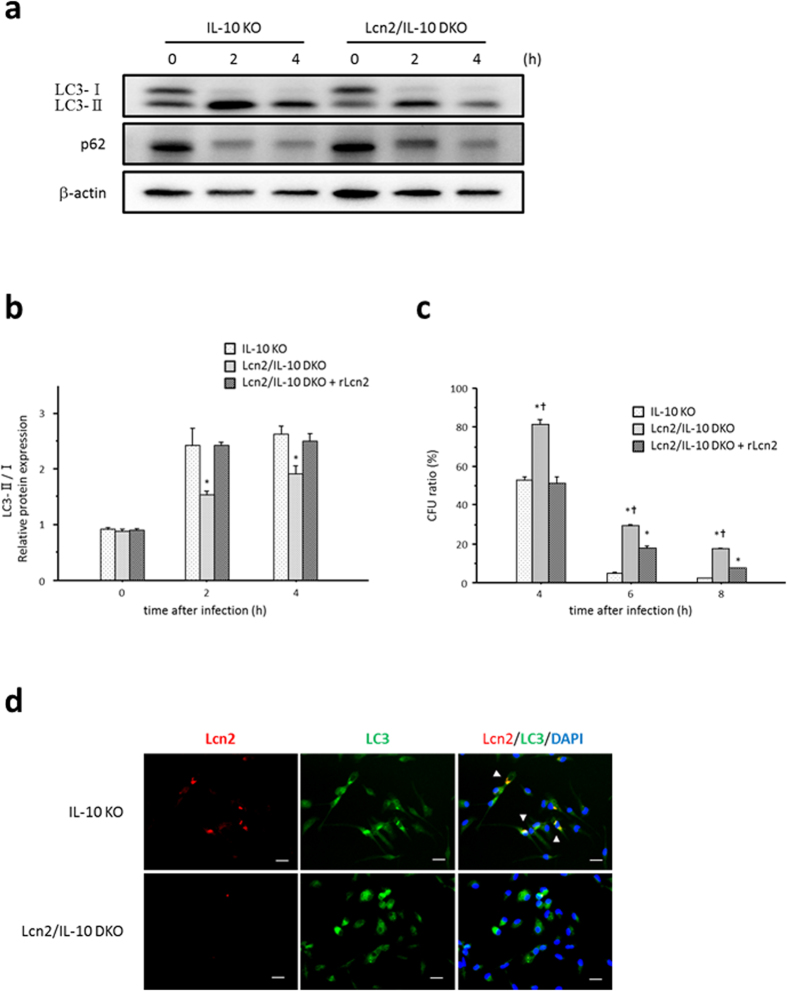
Enhanced phagocytosis/autophagy pathways in macrophages by Lcn2. (**a**) Protein expression of LC3 and p62 in macrophages at 0, 2, and 4 hours after infection with *E. coli*. Thioglycollate-elicited peritoneal macrophages were infected with *E. coli* for 1 hour, and expression of each target protein was evaluated by Western blotting analysis. β-actin is shown as a control. Data shown are representative of each group. (**b**) Quantification of LC3- II protein in macrophages at 0, 2, and 4 hours after infection with *E. coli*. Thioglycollate-elicited peritoneal macrophages obtained from Lcn2/IL-10 DKO mice were pre-incubated with or without 100 ng/ml of recombinant Lcn2 for 4 hours before *E. coli* infection. Expression of LC3 protein was evaluated by Western blotting analysis and is shown as the intensity ratio of LC3- II to LC3- I. N = 6 per group. Error bars represent SEM. *P < 0.05 compared with IL-10 KO mice by one-way ANOVA with Bonferroni’s correction. (**c**) Ratio of colony forming units (CFU) at the indicated times to CFU at 2 hours after *E. coli* infection. N = 6 per group. Error bars represent SEM. *P < 0.05 compared with IL-10 KO mice; ^†^*P* < 0.05 compared to Lcn2/IL-10 DKO mice with recombinant Lcn2 pre-treatment by one-way ANOVA with Bonferroni’s correction. (**d**) Co-localization of Lcn2 and LC3 in macrophages infected with *E. coli*. Double-immunofluorescent staining with anti-Lcn2 and anti-LC3 antibodies was performed in thioglycollate-elicited peritoneal macrophages obtained from IL-10 KO (upper panels) and Lcn2/IL-10 DKO mice (lower panels). Co-localization of Lcn2 and LC3 is indicated by white arrowheads. Data are representative of each group. Scale bars, 20 μm. DAPI, 4′,6-diamidino-2-phenylindole.

The impaired bactericidal activity in DKO-derived TEPMs suggested the involvement of Lcn2 in phagocytosis/autophagy pathways. Therefore, we investigated the effect of Lcn2 deficiency on phagocytosis/autophagy pathways in macrophages by comparing LC3-II formation and p62 degradation between IL-10KO- and DKO-derived TEPMs after *E. coli* infection. LC3-II formation was decreased and p62 expression was increased in DKO-derived TEPMs compared to IL-10KO-derived TEPMs beginning 2 hours after *E. coli* infection (Fig. 6a,b). Pre-incubation with recombinant Lcn2 (rLcn2) restored LC3-II formation in DKO-derived TEPMs after *E. coli* infection (Fig. 6b), resulting in improved bacterial clearance in DKO-derived TEPMs (Fig. 6c). Furthermore, immunocytochemistry showed co-localization of Lcn2 and LC3 in IL-10KO-derived TEPMs 2 hours after infection with *E. coli* (Fig. 6d).

### Lcn2-repleted macrophages prevented the development of colitis in Lcn2/IL-10 DKO mice

**Figure 7:**
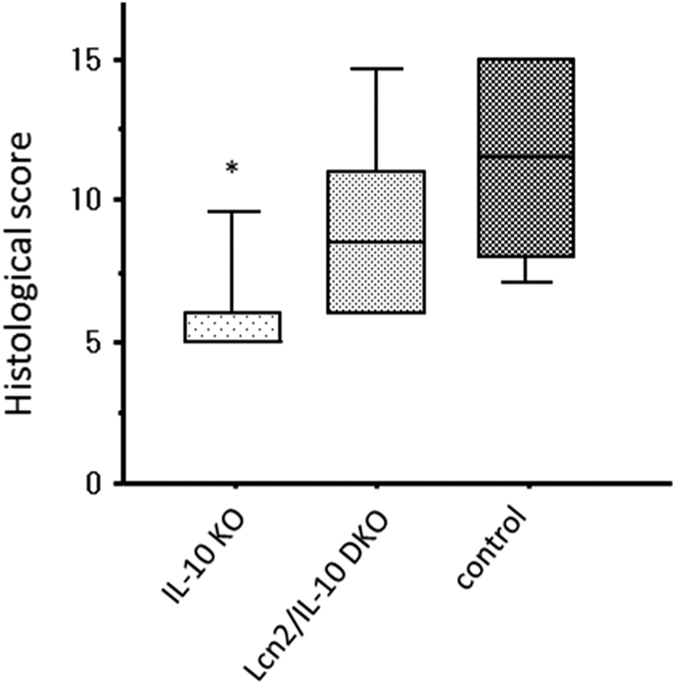
Attenuated colitis in Lcn2/IL-10 DKO mice after transfer of IL-10KO-derived thioglycollate-elicited peritoneal macrophages. Three-week-old Lcn2/IL-10 DKO mice were intraperitoneally injected with thioglycollate-elicited peritoneal macrophages obtained from IL-10 KO or Lcn2/IL-10 DKO mice. In control group, PBS was intraperitoneally injected instead of macrophages. The mice were killed at day 7, and histological inflammation was compared among groups. N = 6 per group. The box refers to the interquartile range and the bar inside represents the median. *P < 0.05 compared to control group by Kruskal-Wallis test with Games-Howell post hoc analysis.

Finally, we performed a macrophage transfer experiment to elucidate the role of macrophage Lcn2 in the development of spontaneous colitis. Transfer of IL-10KO-derived, but not DKO-derived, TEPMs prevented the development of colitis in Lcn2/IL-10 DKO mice and these mice exhibited a significantly lower histological score than control mice at 4 weeks of age (Fig. 7).

## Discussion

The findings of the present study revealed that Lcn2 prevents the development of spontaneous colitis in IL-10 deficient mice by enhancing phagocytic bacterial clearance in macrophages. The results clearly demonstrated a crucial role for Lcn2/NGAL in the development of intestinal inflammation by its regulation of mucosal innate immune responses toward enteric commensal bacteria.

Immunohistochemistry revealed increased Lcn2 expression in the inflamed colonic tissues of IL-10 KO mice. Lcn2 expression was mainly observed in the colonic epithelial cells and partly in the infiltrating immune cells. Also, Østvik *et al*. reported upregulated Lcn2 expression in the colonic epithelia of patients with active IBD compared to patients with inactive IBD by *in situ* hybridisation^17^. Thus, intestinal epithelial cells are the main Lcn2-producing cells in inflamed colonic tissues in both mice and humans. In addition, the fecal concentration of Lcn2 increased in IL-10 KO mice with the development of colitis, thus supporting the notion that Lcn2 is secreted from colonic epithelial cells in response to inflammatory signals.

To determine the type of stimulus that regulates Lcn2 expression in the colon, we stimulated CT26 cells with representative pro-inflammatory cytokines involved in the pathophysiology of IL-10 KO mice^21^,^22^. We also stimulated CT26 cells with ligands for TLR2, TLR4, and TLR9, all of which are expressed by intestinal epithelial cells and recognise enteric bacterial components^23^. These *in vitro* experiments revealed increased Lcn2 production from CT26 cells after stimulation with IL-1β, IL-17A, and LPS. We further confirmed these results *in vivo*, and finally revealed that IL-1β and TLR4 signaling pathways are crucial for enhancing Lcn2 expression in the colonic tissues of IL-10 KO mice. In addition, previous studies using murine tracheal epithelial cells or a human lung epithelial cell-line reported that Lcn2 was upregulated by IL-1β and IL-17A, but not by TNF-α *in vitro*^14^,^24^. Chan *et al*. demonstrated that Lcn2 was induced in IL-1β- and TLR4-dependent manners in the lung after infection with *Klebsiella pneumonia*^14^. On the other hand, Østvik *et al*. reported increased Lcn2 production from human colonic epithelial cell-line, HT-29 cells, after stimulation with IL-1β, but not LPS^17^. These previous findings indicate the critical role of IL-1β and TLR4 signalling pathways for enhancing Lcn2 expression. In contrast to our finding, Cayatte C *et al*. showed significantly reduced Lcn2 expression in colon after challenge with anti-IL-23p19 antibody using murine T-cell transfer colitis model, suggesting the important role of T helper 17 cells-associated cytokines including IL-17A in the regulation of Lcn2 in colon^25^. The conflicting finding might result from the different cytokine profile in the different model of colitis. Stalhofer J *et al*. also showed a synergetic effect of IL-17A with IL-22 and TNF-α for inducing Lcn2 in human colonic epithelial cell lines, and reported that patients with Crohn’s disease carrying IBD risk-increasing *IL23R* alleles who might have impaired T helper 17 cells-associated immune responses showed lower serum Lcn2 concentration than those not carrying^26^. Taken together, further investigation is needed to elucidate the role of IL-17A in the regulation of Lcn2 expression in colon.

We then generated Lcn2/IL-10 DKO mice and compared intestinal inflammation with that of IL-10 KO mice to reveal the role of Lcn2 in the development of colitis. While Lcn2 KO mice did not develop spontaneous colitis, a rapid onset and development of colitis was observed in Lcn2/IL-10 DKO mice soon after the weaning period compared to IL-10 KO mice, suggesting that the innate immune responses were disrupted in the colonic tissues of Lcn2/IL-10 DKO mice. Furthermore, the enhanced Lcn2 production in colonic epithelia in response to inflammatory signals suggests a role for Lcn2 in mucosal barrier function. Therefore, in this study, we focused on the effect of Lcn2 on mucosal defence systems, including mucosal barrier and underlying innate immune cells, especially macrophages. Regarding mucosal barrier function, we first compared the luminal bacterial composition between WT and Lcn2 KO mice by T-RFLP analysis to examine the effect of Lcn2 on enteric bacteria. We also examined the proliferation and differentiation of colonic epithelial cells by counting Ki-67 positive cells in the crypts and quantifying *HES-1* gene expression in the colonic tissues of WT and Lcn2 KO mice^27^. Moreover, we quantified the gene expression of intestinal barrier-related molecules, such as junctional molecules, mucin, trefoil factor, anti-microbial peptides, and TLRs in the colonic tissues of WT and Lcn2 KO mice^23^,^28^. These components, however, did not significantly differ between WT and Lcn2 KO mice, indicating that Lcn2 does not affect mucosal barrier function, at least in non-inflamed colon. A substantial amount of Lcn2 produced in the inflamed milieu, however, might affect mucosal barrier function. In contrast to our results, Singh V *et al*. showed a distinct difference in fecal bacterial composition between WT and Lcn2 KO mice with increased *Bacteroidetes* and *Proteobacteria* phylums and decreased *Tenericutes* phylum in Lcn2 KO mice, although they did not find spontaneous intestinal inflammation in Lcn2 KO mice like our study^29^. Furthermore, they demonstrated the potential colitogenicity of this gut flora of Lcn2 KO mice by co-housing IL-10 KO mice with Lcn2 KO mice. These findings conflicting with our data might result from the different genetic background of mice or environmental factors in the different facility. Further study is necessary to elucidate the effect of Lcn2 on gut microbiota and mucosal barrier function in colon.

Next, we investigated the effect of Lcn2 deficiency on macrophage functions. We used TEPMs in this study, because a recent large-scale genetic analysis of the mouse immune system elucidated the genetic similarity between TEPMs and intestinal macrophages on the basis of macrophage-related gene signatures^30^. We first demonstrated that Lcn2KO-derived TMPMs produced higher mounts of pro-inflammatory cytokines, such as TNF-α and IL-12p40, after stimulation with Pam3CSK4 and LPS, whereas they produced lower amounts of IL-10 compared to WT-derived TEPMs. Zhang *et al*. also reported that pre-treatment with Lcn2 suppressed LPS-induced gene expression of pro-inflammatory cytokines in a murine macrophage cell-line, RAW264.7^31^. Furthermore, Warszawska *et al*. reported significantly higher production of keratinocyte-derived chemokine and lower production of IL-10 in Lcn2KO-derived bone marrow-derived macrophages compared to WT-derived bone marrow-derived macrophages after infection with *Streptococcus pneumonia*^32^. In their study, Lcn2 induced the deactivation of macrophages, characterised by the down-regulation of pro-inflammatory cytokines and the upregulation of anti-inflammatory cytokines, including IL-10. In the present study, however, cytokine production did not differ significantly between IL-10KO- and DKO-derived TEPMs. Thus, the effect of Lcn2 deficiency on cytokine production from macrophages could not be directly involved in the deteriorated colitis of Lcn2/IL-10 DKO mice.

We then assessed the effect of Lcn2 on the phagocytic function of macrophages using pHrodo dye-conjugated *E. coli,* which exhibit a dramatic increase in fluorescence inside the cells. The increased fluorescence intensity in DKO-derived TEPMs compared to other TEPMs after *E. coli* challenge indicated the enhanced uptake of bacteria in DKO-derived TEPMs. Furthermore, we examined the role of Lcn2 in the intracellular bacterial killing function of macrophages using a gentamicin protection assay. Intriguingly, DKO-derived TEPMs showed impaired intracellular bacterial clearance compared to other TEPMs, followed by increased TNF-α production from DKO-derived TEPMs. The increased TNF-α secretion from infected DKO-derived TEPMs, despite no significant difference in TNF-α production between DKO-derived and IL-10KO-derived TEPMs after stimulation with TLR ligands, suggested persistent macrophage activation due to increased numbers of surviving *E. coli* within the cells. In contrast, neither phagocytosis nor intracellular bacterial clearance differed significantly between WT- and Lcn2KO-derived TEPMs. To further explore this discrepancy, we evaluated Lcn2 expression in WT- and IL-10KO-derived TEPMs infected with *E. coli*, and found that IL-10KO-derived TEPMs, but not WT-derived TEPMs, expressed Lcn2 after infection with *E. coli*. Namely, 1-hour infection with *E. coli* might be insufficient for WT-derived TEPMs to express Lcn2, and inadequate Lcn2 expression in WT-derived TEPMs might result in the same level of phagocytosis and intracellular bacterial clearance as Lcn2KO-derived TEPMs.

Autophagy is a cellular degradation system for numerous cytosolic contents, including long-lived proteins, mitochondria, and peroxisomes, and also plays an essential role in the elimination of intracellular bacteria^33^. Furthermore, recent genetic analyses demonstrated an essential role of autophagy in innate immunity, and implicated several autophagy-related genes, such as *ATG16L1* and *IRGM*, in the pathophysiology of IBD^34^,^35^,^36^. Indeed, Lassen *et al*. revealed that Atg16L1 T300A, the coding mutation associated with an increased risk of Crohn’s disease in humans, contributes to decreased antibacterial autophagy and increased IL-1β production from CD11b-positive colonic and splenic macrophages in mice^37^. Induction of autophagy leads to the fusion of bacterial phagosomes with lysosomes facilitated by autophagy-related proteins, such as LC3 and p62, and results in the degradation of bacteria within the formed autolysosome^33^. Therefore, we assessed the effect of Lcn2 deficiency on phagocytosis/autophagy pathways by comparing LC3-II formation and p62 degradation between IL-10- and DKO-derived TEPMs. We also examined the localization of Lcn2 and LC3 in macrophages after infection with *E. coli* by immunocytochemistry. Our data clearly demonstrated that Lcn2 enhances phagocytosis/autophagy pathways in macrophages after infection with *E. coli*. To further confirm the results *in vivo*, we performed a macrophage transfer experiment. Restoring Lcn2 expression in intestinal macrophages by transfer of IL-10KO-derived TEPMs attenuated colitis in 4-week-old Lcn2/IL-10 DKO mice. This finding indicates that Lcn2 in macrophages is crucial for preventing the development of colitis in IL-10 deficient mice, although the incompletely attenuated colitis in IL-10KO-derived TEPMs-transferred Lcn2/IL-10 DKO mice suggested that Lcn2 from other origins, including colonic epithelial cells, could also be involved in the development of colitis. Considering that exogenous Lcn2 enhanced autophagic bacterial clearance in macrophages in our *in vitro* experiments, an intriguing possibility is that Lcn2 secreted from colonic epithelial cells also affects intestinal macrophages to prevent the development of colitis *in vivo*.

In the present study, Lcn2 deficiency enhanced bacterial phagocytosis, but impaired intracellular bacterial clearance in macrophages. Recently, Bonilla *et al*. evaluated the role of autophagy in regulating phagocytosis in macrophages. They demonstrated that p62 accumulation due to impaired autophagy increases the cell-surface expression of class A scavenger receptors, which recognise a diverse group of bacteria, including *E. coli*, leading to enhanced phagocytosis of these bacteria in macrophages^38^. In our study, enhanced uptake of bacteria in DKO-derived TEPMs might attribute to impaired autophagy in DKO-derived TEPMs.

In conclusion, Lcn2 prevented the development of spontaneous colitis in IL-10 deficient mice. One crucial factor seemed to be the enhancement of phagocytic bacterial clearance in macrophages by Lcn2. Further studies of the interaction between Lcn2 and autophagy-related proteins will provide new insight into the role of Lcn2/NGAL in the pathophysiology of IBD.

## Methods

### Ethics statement

This study was performed in strict accordance with the recommendations in the Guide for the Care and Use of Laboratory Animals of the National Institutes of Health. The protocol was approved by the Animal Protection Committee of Kyoto University.

### Mice

C57BL/6 was purchased from Japan SLC (Shizuoka, Japan). B6.129P2-*Il10*^*tm1Cgn*^/J (IL-10 KO) and B6.129P2-*Tlr4*^tm1Aki^ (TLR4 KO) mice were purchased from Jackson Laboratory (Bar Harbor, ME, USA). B6.129P2-*Lcn2*^tm1Aade^/AkiJ (Lcn2) mice were originally generated by Shizuo Akira and provided by Kiyoshi Mori^11^. Lcn2/IL-10 DKO and TLR4/IL-10 DKO mice were generated by intercrossing IL-10 KO mice with Lcn2 KO and TLR4 KO mice, respectively, on C57BL/6 genetic background. Mice were fed standard laboratory chow and supplied drinking water ad libitum. All experiments were performed with female mice at 3 to 12 weeks of age.

### Microscopic assessment of colitis

Mice were killed at 4, 8, and 12 weeks of age. Rectums were dissected and transverse sections prepared. The sections were fixed in 10% formaldehyde, dehydrated in ethanol, and embedded in paraffin for histological analysis. Sections were stained with haematoxylin and eosin (HE), and histologically analysed in a blind manner. Histological damage was quantified by the previously described histological scoring system^39^.

### Immunohistochemistry

Immunostaining was performed as described prevously^39^. Goat anti-mouse Lcn2 antibody, horseradish peroxidase-conjugated donkey anti-goat IgG, and Alexa Fluor 594-conjugated anti-goat antibody were obtained from R&D Systems (Minneapolis, MN, USA), Jackson ImmunoResearch Laboratories (West Grove, PA, USA), and Invitrogen (Carlsbad, CA, USA). The immunofluorescence imaging was acquired using a fluorescence microscope (BIOREVO BZ-9000; Keyence, Osaka, Japan) with the BZ-Analyzer version 2.1 software and composed in Photoshop Elements 12 (Adobe Systems Inc., San Jose, CA, USA).

### Preparation of cecal bacterial lysates

CBLs were prepared from cecal contents of WT mice as described previously^40^ and stored at −20 °C until use. The sterility of the lysates was confirmed by culture.

### Preparation of stool samples

Murine cecal contents were collected using Smart-Prep Fecal Sample Extraction KIT (ALPCO Diagnostics, Salem, NH, USA). Stool samples were subsequently prepared using the extraction buffer from a *PhiCal* Calprotectin ELISA Kit (Immundiagnostik AG, Bensheim, Germany) and stored at −80 °C until analysis.

### Colon fragment culture

Colon fragment culture was performed as previously reported^41^. Culture supernatants were collected and stored at −20 °C until analysis.

### Stimulation of mesenteric lymph node cells

MLN cells were isolated as described previously^39^. Unseparated MLN cells were seeded at 4 × 10^5^ cells/well in 96-well cell culture plates, and incubated in 200 μl of complete RPMI medium (Gibco, Invitrogen, Grand Island, NY, USA) with or without 30 μg/ml CBLs at 37 °C for 72 hours. Culture supernatants were collected and stored at −80 °C until analysis.

### Quantitative analysis of gene expression

Collected rectal tissues were quickly frozen in liquid nitrogen for later mRNA extraction. Total RNA was extracted using the guanidium isothiocyanate-phenol-chloroform method. Extracted RNA was reverse-transcribed with SuperScript II Reverse Transcriptase (Invitrogen) and the resulting complementary DNAs were analysed for the gene expression of target molecules using LightCycler 480 System II (Roche Applied Science, Indianapolis, IN, USA). The primer sequences are shown in Supplementary Table 2. Resulting gene expression levels of target molecules were normalised to the expression of GAPDH.

### Enzyme-linked immunosorbent assay

The Lcn2 levels in stool samples and cell supernatants were quantified using Lcn2 Mouse ELISA kit (R&D Systems) according to the manufacturer’s instructions. The TNF-α, IL-12p40, IL-10, IFN-γ, and IL-17A levels in the supernatants of the culture medium were quantified using a Mouse ELISA kit (eBioscience, San Diego, CA, USA) according to the manufacturer’s instructions.

### Cell culture

The murine intestinal epithelial cell line CT26 (ATCC; CRL-2638) was used. CT26 cells were cultured with complete RPMI medium containing 10% heat-inactivated foetal bovine serum, 100 mg/ml streptomycin, and 100 mg/ml penicillin. Cells were incubated in a 5% CO_2_ incubator at 37 °C. For stimulation trials, CT26 cells were seeded at 1 × 10^6^ cells/well in 6-well culture plates. The stimulators were added as follows: Pam3CSK4 (TLR2 ligand) 1 μg/ml, LPS (TLR4 ligand) 100 ng/ml, ODN1585 (TLR9 ligand) 100 nM, IFN-γ 10 ng/ml, TNF-α 100 ng/ml, IL-1β 10 ng/ml, and IL-17A 200 ng/ml (Pam3CSK4 and ODN1585 from InvivoGen [San Diego, CA, USA], LPS from Sigma-Aldrich [Saint Louis, MO, USA], and others from R&D Systems). Cells were stimulated for 24 hours before supernatants were harvested, and stored at −80 °C.

### Administration of neutralizing antibodies

The neutralizing goat anti-mouse IL-1β, rat anti-mouse IL-17, and their control antibodies (goat IgG control and rat IgG2a isotype control) were purchased from R&D Systems. Three-week-old IL-10 KO mice were intraperitoneally injected with 100 μg of anti-IL-1β or goat control antibody on day 0^42^. Otherwise, 100 μg of anti-IL-17 or rat control antibody was intraperitoneally injected on days 0, 2, 4, and 6^43^. The mice were killed on day 7 and fecal proteins were extracted as described above.

### Terminal restriction fragment-length polymorphism analysis

T-RFLP analysis was performed as described previously^44^. The 16S rRNA gene was amplified from fecal DNA with labelled primers and treated with 10 U *Bsl* l (New England BioLabs, MA, USA). The digested products were fractionated using an ABI PRISM 3130xl Genetic Analyzer (Applied Biosystems, CA, USA) with DNA analysis software Gene Mapper. The major terminal restriction fragments of 1~3 bp were summarized as operational taxonomic units, and the corresponding bacterial groups were estimated by computer simulation based on a human intestinal microbiota database^45^. The percentage of each bacterial group to the total enteric bacteria was represented by the percentage of each summed operational taxonomic unit area to total operational taxonomic unit area^46^.

### Preparation of thioglycollate-elicited peritoneal macrophages

Mice were intraperitoneally injected with 4% thioglycollate (Eilken Chemical Co., Tokyo, Japan) and peritoneal cells were harvested on day 4. The TEPMs were prepared after removing erythrocytes with lysis buffer. In several experiments, the TEPMs were seeded at 1 × 10^6^ cells/well in 6-well culture plates and incubated overnight in complete RPMI medium containing 40 ng/ml recombinant macrophage colony-stimulating factor (rM-CSF; Peprotech, Rocky Hill, NJ, USA) before stimulating with Pam3CSK4 (500 ng/ml), LPS (100 ng/ml), or ODN1585 (500 nM) for 24 hours. Supernatants were harvested and stored at −80 °C until analysis.

### Phagocytosis assay

The phagocytosis assay was performed using pHrodo Green *E. coli* BioParticles Conjugate (Invitrogen) according to the manufacturer’s instructions. The fluorescence intensity was measured at the indicated times using a fluorescence microplate reader (Infinite F200 PRO; TECAN, Kawasaki, Japan). Phagocytic activity was evaluated by subtracting the mean fluorescence intensity of no-cell negative-control wells from all experimental wells and is indicated as relative value.

### Gentamicin protection assay

A gentamicin protection assay was performed as previously described with some modifications^47^. Briefly, TEPMs were seeded at 5 × 10^5^ cells/well in 12-well cell culture plates and incubated overnight in complete RPMI medium containing 40 ng/ml rM-CSF. The wells were challenged with *E. coli* (ATCC; 25922) at a multiplicity of inoculation of 10 bacteria per cell. In several experiments, cells were further incubated with 100 ng/ml recombinant Lcn2 (R&D Systems) for 4 hours before challenge with *E. coli*. Cells were incubated for 1 hour to internalise the bacteria, washed with phosphate buffered saline (PBS), and cultured in complete RPMI medium containing 100 mg/ml gentamicin. At several time points after infection, the cells were lysed with 1% Triton-X 100 in PBS, and surviving bacteria were counted on Brain-Heart Infusion agar plates. Bactericidal activity of macrophages was evaluated by calculating the ratio of colony forming units at each time point to that at 2 hours after infection.

### Western blot analysis

Western blot analyses were performed on whole-cell extracts as described previously^40^. Rabbit anti-mouse LC3, p62, and β-actin were obtained from Cell Signaling Technology (Beverly, MA, USA). The image was recorded using a chemiluminescent image reader (ChemiDoc XRS plus, Bio-Rad Laboratories).

### Immunocytochemistry

TEPMs were seeded on culture cover glasses (Matsunami Glass, Osaka, Japan) and incubated overnight. After infection with *E. coli*, the cells were washed with PBS and fixed with 4% paraformaldehyde at 4 °C overnight. For antigen retrieval, the cells were boiled in citrate buffer (pH 6.0) in a microwave oven for 10 minutes. After blocking with 5% BSA in PBS containing 0.1% Tween20 (PBS-T), cells were incubated with goat anti-Lcn2 antibody and rabbit anti-mouse LC3 for 1 hour at room temperature. After washing with PBS-T, cells were incubated for 30 minutes with Alexa Fluor 488-conjugated anti-rabbit antibody (Invitrogen) and Alexa Fluor 594-conjugated anti-goat antibody. Immunofluorescence imaging was acquired using a fluorescence microscope (BIOREVO BZ-9000) with BZ-Analyzer version 2.1 software and composed in Photoshop Elements 12.

### Macrophage transfer

Macrophage transfer was performed as described previously with some modification^48^,^49^. Briefly, 3 × 10^7^ TEPMs were prepared from IL-10 KO or Lcn2/IL-10 DKO mice and suspended in 300 μl PBS. Three-week-old mice were intraperitoneally injected with TEPM, or PBS as a control. The mice were killed at day 7, and histological inflammation was evaluated as described above.

### Statistical analysis

Numerical data are expressed as means with SD or SEM, and the differences between groups were analyzed by one-way analysis of variance (ANOVA) with Bonferroni’s correction for multiple comparisons if variables distribute normally. Numerical data are expressed as medians with interquartile ranges, and the differences between groups were analyzed by non-parametric Mann-Whitney U test or Kruskal-Wallis test with Games-Howell post hoc analysis for multiple comparisons if variables do not distribute normally. A *P* value of less than 0.05 was considered statistically significant.

## Additional Information

**How to cite this article**: Toyonaga, T. *et al*. Lipocalin 2 prevents intestinal inflammation by enhancing phagocytic bacterial clearance in macrophages. *Sci. Rep.* **6**, 35014; doi: 10.1038/srep35014 (2016).

## Supplementary Material

Supplementary Information
