# Supplementary material for: Lipocalin 2 prevents intestinal inflammation by enhancing phagocytic bacterial clearance in macrophages
Source: Sci Rep. 2016 Oct 13;6:35014. doi: 10.1038/srep35014 (PMC5062163; doi:10.1038/srep35014)
Supplement: Supplementary Information [file srep35014-s1.pdf]

Lipocalin 2 prevents intestinal inflammation by enhancing phagocytic bacterial clearance in macrophages

Takahiko Toyonaga[1], Minoru Matsuura[2], Kiyoshi Mori[3], Yusuke Honzawa[2], Naoki Minami[2], Satoshi Yamada[2], Taku Kobayashi[1], Toshifumi Hibi[1], Hiroshi Nakase[4★]

Supplementary Figure 1

Lcn2 KO mice did not develop spontaneous colitis.

Representative HE-staining images of colonic tissues from Lcn2 KO mice at the indicated age (in weeks). Scale bars, 100 µm. wk, week of age.

Supplementary Figure 2

Lcn2 deficiency did not affect mucosal barrier function.

(**a**) T-RFLP profiles of faecal samples from WT and Lcn2 KO mice at 4 weeks of age. N = 6 per group.

(**b**) Left, immunohistochemical staining for Ki-67 in the colonic tissues of WT and Lcn2 KO mice. Representative images are shown for each group. Right, number of Ki-67 positive colonic epithelial cells in the crypts of WT and Lcn2 KO mice. Ki-67 positive cells were counted in 20 crypts per mouse. N = 3 per group.

(**c-d**) Gene expression of *HES-1* (**c**) and intestinal barrier-related molecules (**d**) in the colonic tissues of WT and Lcn2 KO mice. Gene expression of each target molecule was normalised to *GAPDH*. N = 6-8 for each group. The box refers to the interquartile range and the bar inside represents the median. Statistical significance was analysed by non-parametric Mann-Whitney U test. *N.S.*, not significant

Supplementary Figure 3

Protein expression of Lcn2 in macrophages after infection with *E. coli*.

Thioglycollate-elicited peritoneal macrophages were infected with *E. coli* for 1 hour. Lcn2 expression was evaluated by Western blotting analysis at 0, 2, 4, and 6 hours after infection.

Supplementary Fig.1

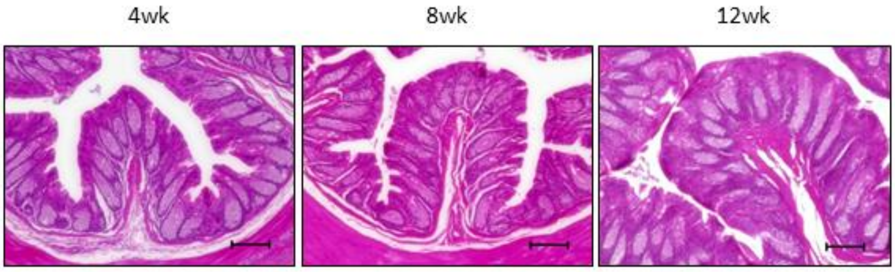

Supplementary Fig.2

a

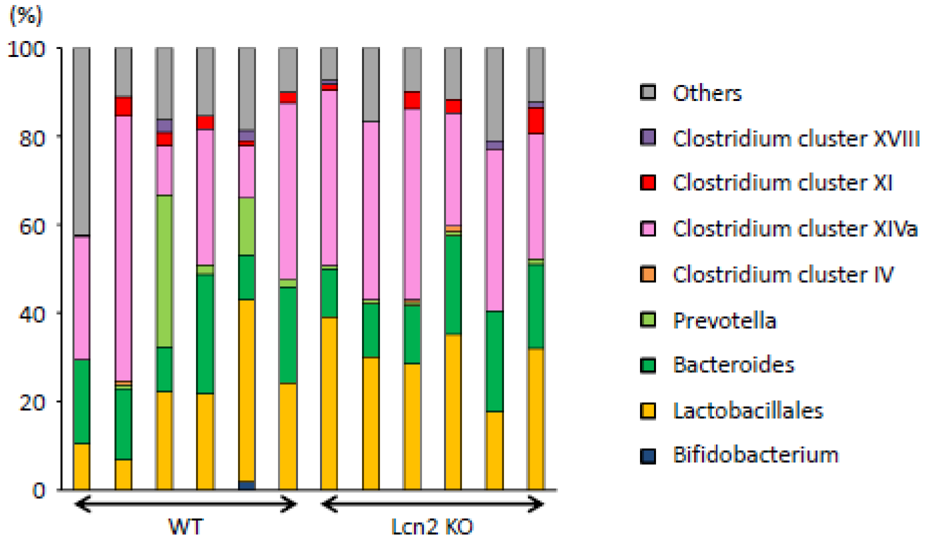

b

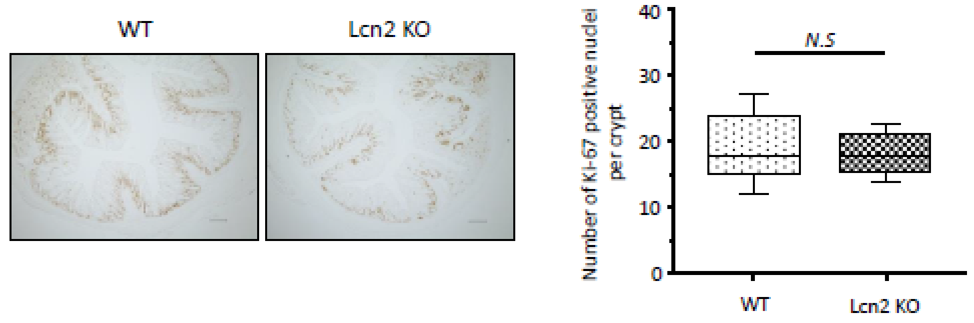

c

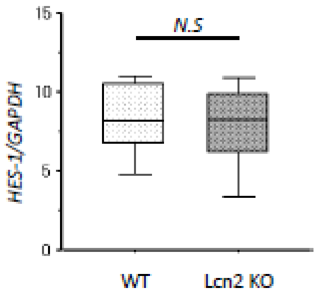

Supplementary Fig.2

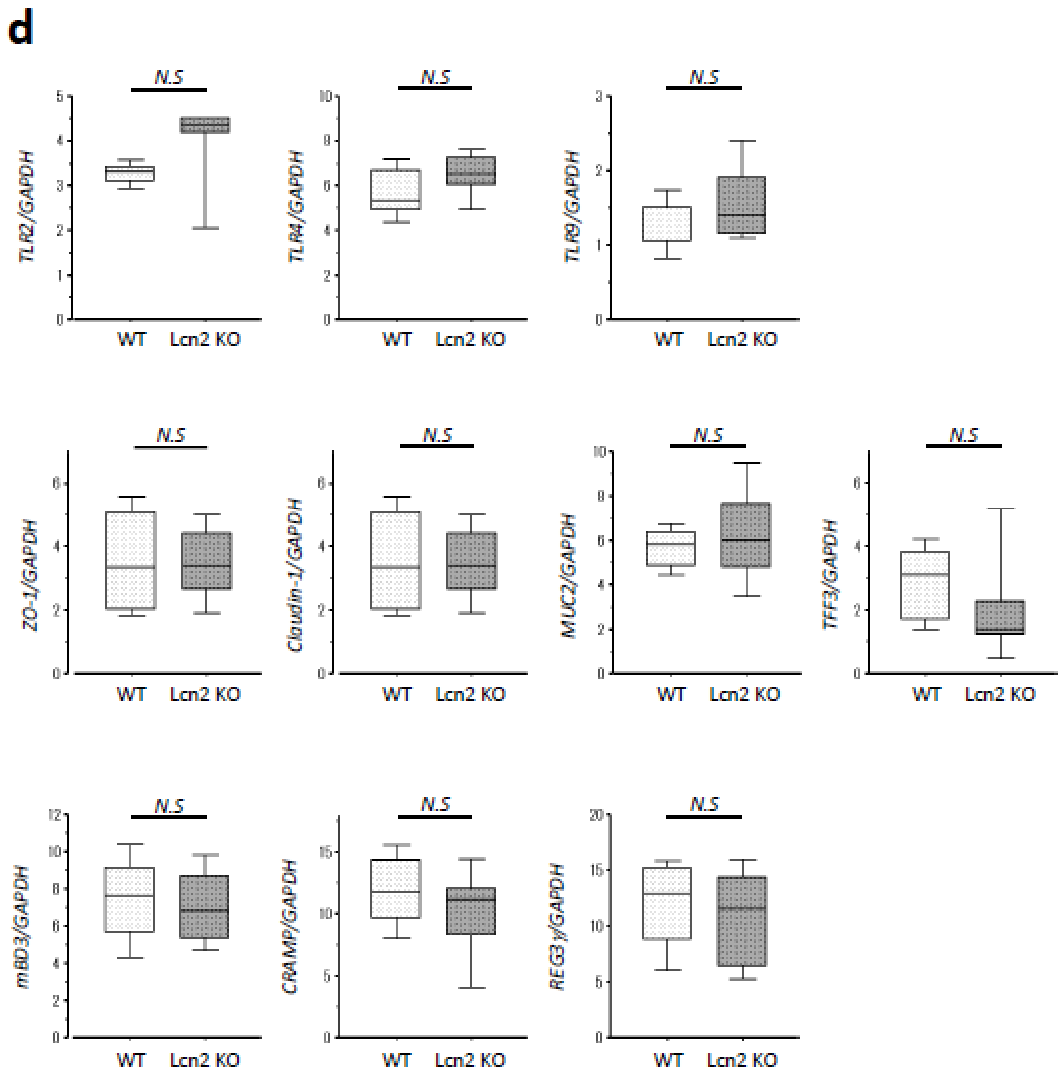

Supplementary Fig.3

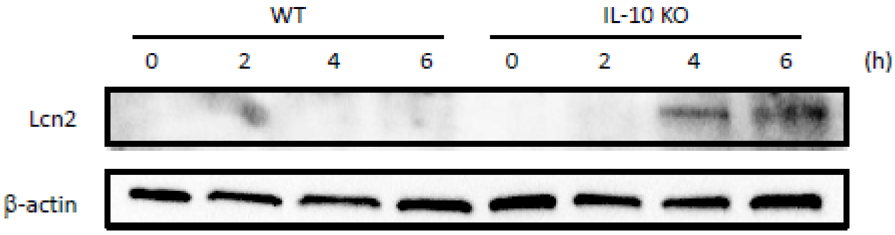

Supplementary Table 1. T-RFLP profiles of fecal samples from WT and Lcn2 KO mice

Predicted bacteria	WT	Lcn2 KO	*P* values
Bifidobacterium	0.0 (0.0-0.6)	0.0 (0.0-0.0)	0.1396
Lactobacillales	21.9 (9.6-28.5)	31.1 (25.8-36.3)	0.1495
Bacteroides	17.5 (10.1-23.0)	16.1 (11.7-22.3)	0.8728
Prevotella	1.9 (0.7-18.4)	0.8 (0.5-1.0)	0.0766
Clostridium cluster IV	0.0 (0.0-0.2)	0.0 (0.0-0.7)	0.5283
Clostridium subcluster XIVa	29.2 (11.6-45.0)	38.1 (27.6-40.8)	0.4704
Clostridium cluster XI	2.6 (0.9-3.4)	2.4 (0.0-4.4)	0.8719
Clostridium cluster XVIII	0.2 (0.0-2.4)	0.5 (0.0-1.3)	0.7976
Others	16.0 (10.9-24.7)	12.1 (9.3-18.0)	0.4233

Each value indicates the percentage of individual predicted bacteria to the total enteric bacteria. N = 6 per group.

Values are expressed as medians with interquartile ranges in parentheses. *P* values were determined by non-parametric Mann-Whitney U test.

Supplementary Table 2. Primer sequences for quantitative PCR

Target gene	Primer sequences	
Gapdh	Forward:	5’-CATGGCCTTCCGTGTTCCTA-3’
	Reverse:	5’-GCGGCACGTCAGATCCA-3’
Lcn2	Forward:	5’-CCATCTATGAGCTACAAGAGAACAA-3’
	Reverse:	5’-CCTGTGCATATTTCCCAGAGTGA-3’
Il1b	Forward:	5’-CAACCAACAAGTGATATTCTCCATG-3’
	Reverse:	5’-GATCCACACTCTCCAGCTGCA-3’
Il17a	Forward:	5’-TCCAGAAGGCCCTCAGACTAC-3’
	Reverse:	5’-ATTGACACAGCGCTGGTGGC-3’
Ifng	Forward:	5’-TCTTCAGCAACAGCAAGGCG-3’
	Reverse:	5’-AACAGCTGGTGGACCACTCG-3’
Tnf	Forward:	5’-CATGCACCACCATCAAGGAC-3’
	Reverse:	5’-GGCCTGAGATCTTATCCAGCC-3’
Muc2	Forward:	5’-GTGCCTAGTCCTGGCCTTAG-3’
	Reverse:	5’-AATCGGTAGACATCGCCGTC-3’
Tff3	Forward:	5’-GCAGTGGTCCTGAAGCTTGC-3’
	Reverse:	5’-TGGCTTGGAGACAGGCCAAC-3’
Defb3	Forward:	5’-GGCTTCAGTCATGAGGATCC-3’
	Reverse:	5’-CCAATGCACCGATTCCAGCATC-3’
Camp	Forward:	5’-ATGCTGTGCTCCGAGCTGTG-3’
	Reverse:	5’-TCACTCGGAACCTCACAGAC-3’
Reg3g	Forward:	5’-CTTGTGTCTGTGCTCAGTGG-3’
	Reverse:	5’-GGTTCATAGCCCAGTGTCGG-3’
Tjp1	Forward:	5’-CGAAACTGATGCTGTGGATAG-3’
	Reverse:	5’-TGATACTGAGTTGCCTTCACC-3’
Cldn1	Forward:	5’-TCTGCATCTGCCACTGAGCC-3’
	Reverse:	5’-GAGCGGTCACGATGTTGTCC-3’
Tlr2	Forward:	5’-CGTCTTGGAATGTCACCAGG-3’
	Reverse:	5’-CCACAGTCCGTGGAAATGGT-3’
Tlr4	Forward:	5’-ATTCCTGGTGTAGCCATTGC-3’
	Reverse:	5’-AAGATACACCAACGGCTCTG-3’
Tlr9	Forward:	5’-ATTCTCAAGACGGTGGATCG-3’
	Reverse:	5’-AGTAAGTCTACGAAGGCTGC-3’
Hes1	Forward:	5’-CTTCAGCGAGTGCATGAACG-3’
	Reverse:	5’-AGGTCATGGCGTTGATCTGG-3’
